# Hybrid of Deep Learning and Word Embedding in Generating Captions: Image-Captioning Solution for Geological Rock Images

**DOI:** 10.3390/jimaging8110294

**Published:** 2022-10-22

**Authors:** Agus Nursikuwagus, Rinaldi Munir, Masayu Leylia Khodra

**Affiliations:** 1 Doctoral Program of Informatics, School of Electrical Engineering and Informatics, Institut Teknologi Bandung, Jl. Ganesha No.10, Bandung 40132, Indonesia; 2 Department of Informatics, School of Electrical Engineering and Informatics, Institut Teknologi Bandung, Jl. Ganesha No.10, Bandung 40132, Indonesia

**Keywords:** deep learning, vector embedding, convolutional neural network, recurrent neural network

## Abstract

Captioning is the process of assembling a description for an image. Previous research on captioning has usually focused on foreground objects. In captioning concepts, there are two main objects for discussion: background object and foreground object. In contrast to the previous image-captioning research, generating captions from the geological images of rocks is more focused on the background of the images. This study proposed image captioning using a convolutional neural network, long short-term memory, and word2vec to generate words from the image. The proposed model was constructed by a convolutional neural network (CNN), long short-term memory (LSTM), and word2vec and gave a dense output of 256 units. To make it properly grammatical, a sequence of predicted words was reconstructed into a sentence by the beam search algorithm with K = 3. An evaluation of the pre-trained baseline model VGG16 and our proposed CNN-A, CNN-B, CNN-C, and CNN-D models used BLEU score methods for the N-gram. The BLEU scores achieved for BLEU-1 using these models were 0.5515, 0.6463, 0.7012, 0.7620, and 0.5620, respectively. BLEU-2 showed scores of 0.6048, 0.6507, 0.7083, 0.8756, and 0.6578, respectively. BLEU-3 performed with scores of 0.6414, 0.6892, 0.7312, 0.8861, and 0.7307, respectively. Finally, BLEU-4 had scores of 0.6526, 0.6504, 0.7345, 0.8250, and 0.7537, respectively. Our CNN-C model outperformed the other models, especially the baseline model. Furthermore, there are several future challenges in studying captions, such as geological sentence structure, geological sentence phrase, and constructing words by a geological tagger.

## 1. Introduction

Geological observation involves field research by a geologist. One of the tasks is to write about the rock’s content and take a photo of the rock. Each picture is paired with its description. This task requires an expert to write carefully and accurately about each image. Each description should contain the rock’s characteristics, including the rock’s color, shape, and constituents. This process is an essential task because the information helps in decisions on other activities, such as mining, land fertilization, field surveillance, and drilling. Sometimes, a geologist finds the same characteristics in the rocks that correspond to previous descriptions and repeatedly writes the same descriptions. It is interesting to adopt these tasks for researching how to make descriptions for the content of a photo image. The geologist’s experience can be assumed as staging for the creation of a description for each rock image. In computer vision terms, those activities can be defined as captioning. One challenging caption process is how to make the images and their descriptions pairwise. With captioning in place, we can predict and describe the content of other photos. Adopting a geologist’s knowledge as an intelligent system for captioning is one aspect that can be explored. How to identify rocks and create descriptions of the content of rock images has been proposed as a research topic.

Most works have proposed some image-captioning concepts. Krizhevsky proposed a CNN for ImageNet and contributed to image extractions [1]. Other research proposed by Karpathy introduced some concepts to make a caption from an image [2]. Supported by ImageNet classification, Karpathy successfully contributed to creating a captioning model for MS COCO and Flickr images [2]. The foreground object is one focus for identifying the objects in an image; the words are then joined to make a sentence that the reader can understand. Ignoring the background object should be avoided when generating a caption. The same works have contributed different ideas, such as captioning based on an identified word-by-word phrase between an object and other objects [3]. Captioning based on text analysis has also been proposed [4,5,6,7,8,9]. Other concepts, such as identifying objects by the object element [10,11,12,13,14,15,16] and studying the generation of captions as part of speech guidance, have been proposed by [8,9,17,18,19,20,21,22,23].

The development of the concept of image captioning cannot be separated from the development of deep learning models. The convolutional neural network (CNN) and recurrent neural network (RNN) can predict the word that pairs with the image region and the predicted word is close to a human description. The CNN, developed by Lecun, has supported production feature images by approximating the classification of the image [24,25]. On the other hand, the RNN also helps generate words that can be paired with the image region, followed by the conditional probability formulation of $L\left(x\right)=p(x\;|x_{1}\ldots x_{i-1};I)$ [26,27,28,29,30,31,32,33,34,35,36]. Model image captioning has been researched by many using CNN block and language models, such as DenseNet and LSTM [9,17], CNN and LSTM [19,26,33,37,38,39], inceptionV3 and RNN [14], and CNN and BERT [40,41]. One of the important parts of captioning is word embedding, which provides a vector feature value for each word. Word2vec [2,6,18,42,43,44] and one-hot-vector [20,23,34,44,45,46,47] are embedding models that many scholars have used for image captioning.

Many research studies have introduced a captioning model that strengthens a foreground object [5,11,12,14,15,16,18,20,23,36,37,38,44,46,48,49,50,51,52,53,54,55,56]. Images contain two parts: foreground and background objects. Foreground objects are identified by making a class of objects, such as cars, men, women, roads, stairs, grass, birds, and animals, whereas a *background object* is an area lying behind the foreground objects at the back of the image, including objects, such as walls, yards, and the sky. In a geological image, the rock is a dominant background object presented at the back of the image [57,58,59,60].

The problem with this study was how to identify the object that appears behind the more common objects. Previous research has not focused on the background of common objects. Thus, it is a challenge to research and explore this domain, especially with geological rock images. Many researchers have proposed effective captioning models. Nevertheless, the resulting BLEU (B@N) scores have not achieved a desirable score. Moreover, the geological rock captions have not proven similar to the geologist’s descriptions. A preliminary experiment performed using CNN + LSTM + one hot vector only presented scores of 0.3990, 0.3830, 0.4050, and 0.3470 for B@1, B@2, B@3, and B@4, respectively [2]. The model identified common objects, such as humans, cars, and animals. All background objects, including the rock, were unnecessary to infer a caption. These results prove that there are still challenges in captioning.

**Figure 1:**
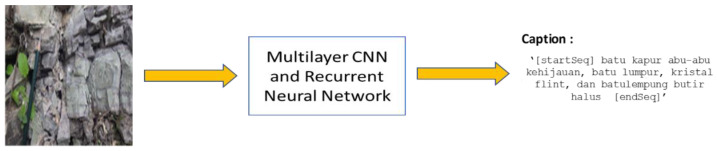
Research Problem on captioning geological terms. The translation of the caption is greenish-gray limestone, mudstone, flint crystal, and fine-grain claystone (CNN: convolutional neural network).

The interpretation of objects from the geologist’s perception encompasses all objects that appear in the background of the image visualization. The geologist focuses on interpreting the rock in the image and ignores the common objects. This is contrary to the previous studies about captioning and a different way of presenting captions. Captioning experiments from this perspective is directed to describing and identifying the rock in the images. Other aspects in the images, such as people, cars, and animals, are not important for identifying the rock. Furthermore, our model can predict the words for the rock in the image and acquire the image’s caption. Figure 1 highlights the problem domain of the study. The objective was to generate a caption for rock images. In line with the research objective, the caption outcomes are in Indonesian and match with the image of the rock.

With regard to the problem and the proposed model, we propose contributions to solve some problems relating to captioning. Our contribution can be presented as follows:

- Using geological field exploration to support captioning and build a model that produces a caption from an image. We collected that geological knowledge and used it to construct an algorithm and the architecture of the captioning model.
- Building the corpus for captioning that contains the pairwise images of rocks and their captions.
- Building a captioning model that can interpret images of rocks and achieve outcomes with an accuracy that is similar to a geologist’s annotation. Our models can outperform the baseline model relating to the BLEU score and acquire captions that are similar to a geologist’s annotation.

We arranged the sections in this paper as follows. The paper leads with an introduction, which encompasses the urgency for this type of research and the research problems. The methods section details content theory and related research, with associated research, such as on convolutional neural networks, long short-term memory, BLEU score measurement, and the estimation function. The subsequent section presents the proposed model to solve the current problems. This paper also conveys the outcome of experiments using the proposed model. Then, the discussion section explains the results. The last section presents the conclusions.

## 2. Methods

### 2.1. Long Short-Term Memory (LSTM)

The LSTM language model can be used as part of the process of generating captions automatically. Another method of sentence representation is the RNN, a simpler method than LSTM. Karpathy used the same number of dimensions as the length of words in the image descriptions from the experts [2]. The RNN method is a simple approach for producing the sentence representation of the detected object. This approach does not consider the order or context of information in a sentence. In the sequence, the results obtained are not grammatically arranged. Karpathy used bi-gram techniques or two dependency relations to generate a sentence with support from either the beam search algorithm or the greedy search algorithm to overcome this [2].

**Figure 2:**
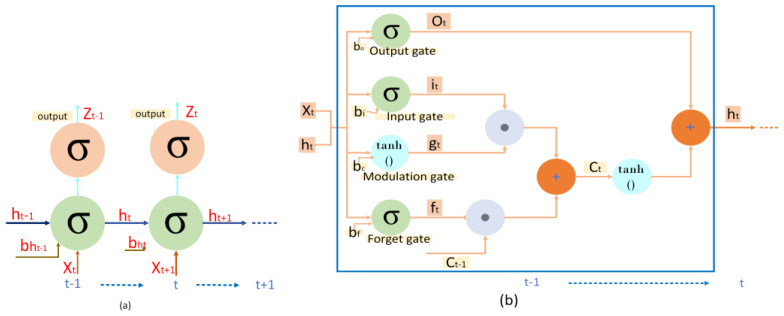
(**a**) RNN and (**b**) LSTM decoder architecture (RNN: recurrent neural network, LSTM: long short-term memory).

Long short-term memory (LSTM) is a language model, the successor of the RNN, that enables long-term learning. The LSTM unit has an additional hidden state as a nonlinear mechanism that allows a state to perform a backpropagation process without modification, change, or reset. Learning in LSTM uses simple function gates to learn speech recognition and language translation [19]. Figure 2 is a simple form of the recurrent neural network (RNN) and LSTM. LSTM is processed by repeating each process performed. In LSTM, the value $\sigma \left(x\right)={\left(1+e^{-x}\right)}^{-1}$ as a sigmoid function with an accurate boundary value between 0 and 1, whereas for nonlinear hyperbolic functions $tanh\left(x\right)=\frac{e^{x}-e^{-x}}{e^{x}+e^{-x}}=2\sigma \left(2x\right)-1$ with input range values between −1 and 1. 

LSTM updates based on time *t* for input $x_{t}$, $h_{t-1}$, and $c_{t-1}$ following the functions below:

(1) $$i_{t}=\sigma \left(W_{xi}x_{t}+W_{hi}h_{t-1}+b_{i}\right)$$

(2) $$f_{t}=\sigma \left(W_{xf}x_{t}+W_{hf}h_{t-1}+b_{f}\right)$$

(3) $$o_{t}=\sigma \left(W_{xo}x_{t}+W_{ho}h_{t-1}+b_{o}\right)$$

(4) $$g_{t}=tanh\left(W_{xc}x_{t}+W_{hc}h_{t-1}+b_{c}\right)$$

(5) $$c_{t}=f_{t}\;{}^{\circ}\;c_{t-1}+i_{t}{}^{\circ}g_{t}$$

(6) $$h_{t}=o_{t}{}^{\circ}tanh\left(c_{t}\right)$$

where $i_{t}$ stands for input at timestamp *t*, $f_{t}$ is the symbol for the forget gate at timestamp *t*, $o_{t}$ stands for the output gate at timestamp *t*, and $g_{t}$ is a hyperbolic function, $c_{t}$ stands for the concatenation elementwise operation, and $h_{t}$ is an output state. 

The RNN updates the values on time *t* for input $x_{t}$ and $h_{t-1}$ following the formulas below:

(7) $$h_{t}=g\left(W_{xh}x_{t}+W_{hh}h_{t-1}+b_{h}\right)$$

(8) $$\;z_{t}=g\left(W_{hz}h_{t}+b_{z}\right)$$

where *g* is an elementwise nonlinearity, such as a sigmoid function or hyperbolic tangent, $x_{t}$ is an input, $h_{t}$ ∈ ${\mathbb{R}}^{N}$ is a hidden state with N hidden units, and $z_{t}$ is the output at time *t*. For the length of the T input sequence $x_{1},\;x_{2},\ldots ,x_{T}$, the update carried out is to calculate sequentially by ignoring $h_{0},$ $h_{1},z_{1},h_{2},z_{2},\ldots ,h_{T},z_{T}.$

### 2.2. Part One Architectures

Our pipeline model was constructed and strengthened by: (1) image preprocessing, including resizing, reducing, and cropping; (2) ensuring that the image could be recognized without losing the pixel information when using the reduce function in the CNN; (3) finding a suitable CNN for our domain, considering CNN layers, dropout, pooling, and dense units. Captioning architecture is essential to generate a word approximate to the human description. Identifying the rocks in the image differs with regard to the colors in the image. 

**Figure 3:**
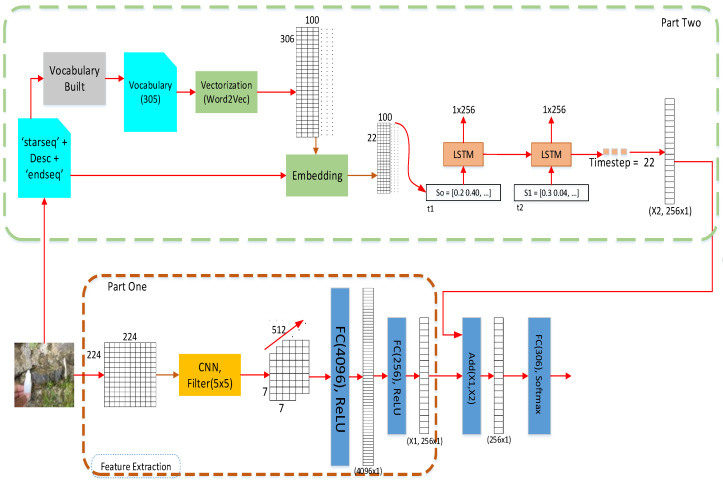
Proposed deep learning architecture (CNN: convolutional neural network, LSTM: long short-term memory).

Figure 3 depicts a geology caption model and divides the model into parts, such as image extraction, word embedding, generating words, and assembling the captions. The image extraction part is in part one and word embedding is in part two. The outcome units are 256 units for each learning stage. We concatenated both outcomes between image extraction and the LSTM unit. Furthermore, after compiling using an ADAM optimizer with learning = 0.0001, we acquired 12,746,112, 2,397,504, 20,482,432, and 104,867,300 training parameters for CNN-A, CNN-B, CNN-C, and CNN-D, respectively. These parameters were achieved from the reengineering of VGG16 and word embedding [2].

Following the significant step, the captioning model always starts with a feature extraction model. An input image had a size of 224 × 224 with RGB color and three channels. We worked on the recognized image to convert it into an RGB value with three channels. After that, we proposed the image extracts using a CNN, shown in Figure 3 [52]. *A*rchitecture classification *is the classification model that identifi*es every single rock [53]. The output of the convolution process was dense with 4096 units. The convolution utilized the dropout function to avoid overfitting, with a rate of 0.5. Every value in a feature map was scaled up using the formula x/(1–0.5), where x is the single feature map. 

After the dropout function, the process continued to leverage linear activation or ReLU and gave outcomes of 256 units [17]. The ReLu function calculated a maximized output between the vector feature value and 0 or Max[x,0]. The ReLu function will present 0 if the feature value is smaller than 0 or the original, x. The outcome of the ReLu function was 256 units. The outcome of part one was a sequence unit with a length of 256 neuron units.

The pipeline process of part one can be written as the following pseudocode:

1. Input Image (*i*_1_, *i*_2_, *i*_3_,…, *i_n_*)—*n* stands for collecting the image, *i*;
2. Reduce image; function for reduction of image to a size of 224 × 224;
3. For *I* $\in \left(i_{1},i_{2},i_{3},\ldots ,i_{n}\right)$—*I* is the collection of the image:
  - Image_feature = CNN(I, C, F)—I is the image with a size of 224 × 224; C represents the channels of convolutions; F is the filter matrix size that can be 3 × 3 or 5 × 5;
  - Feature_Pooling = MaxPooling(Image_feature)—operation of the MaxPooling function;
  - Feature_ReLu = ReLu Activation (Feature Pooling)—run of the ReLu activation;
  - Feature_Dense = Dense(Feature_ReLu)—provides outcome units with 256 dense units.
4. Return (Feature_Dense).

### 2.3. Part Two Architectures

In Figure 3, part two is a concept to create a vector feature value from a geologist’s description. Word2vec is an important embedding model that provides a vector sequence value for each vocabulary unit. Word2vec can produce a vector with 306 × 100 dimensions for word embedding [54]. After the embedding process, vector word embedding was transformed into a vector feature for 22 words for captions that had 22 × 100 dimensions. LSTM used those vectors to generate a word that matched the image feature. Furthermore, the process continued to the dense layer to obtain the Max value via the ReLu function. The last process in part two was to carry out an output of 256 units. This study used the SoftMax, Equation (14), function to acquire a probability value and select the higher probability as a proper word. Equation (14) is a SoftMax function $\sigma \;:\;{\mathbb{R}}^{K}\;\to {\left[0,1\right]}^{K}$ which is defined as follows:

(9) $$\sigma {\left(z\right)}_{i}=\frac{e^{z_{i}}}{\sum_{j=1}^{K}e^{z_{j}}}$$

where *i* = 1 … *K* and *z* = $\left(z_{i}\ldots z_{K}\right)\;\in {\mathbb{R}}^{K}$; $\sigma {\left(z\right)}_{i}$ is a probability value for every unit at index-i; and $e^{z_{i}}$ is an epsilon of the vector at each unit, *z*, at index-i. 

We proposed the process flow of part two from our architectures. This pseudocode writes the following schema of process:

1. C = Input (caption)—input the corpus from the geologist’s annotation;
2. X = ‘start_seq’—initialization of the word;
3. U = unique_word(C)—building a unique word into a vocabulary that attaches from the corpus, C;
4. C_Index = Making_Index(C)—providing the index for each word of the vocabulary;
5. C_vector = Word2Vec(C_Index)—achieving the feature vector from the pre-trained model. The outcomes were 306 × 100 dimensions;
6. W_embedding = Embedding (Vector word ‘Start_seq’ + C_vector);
7. Word_predict = LSTM (w_embedding);
8. Return (feature Length = 256 units).

**Figure 4:**
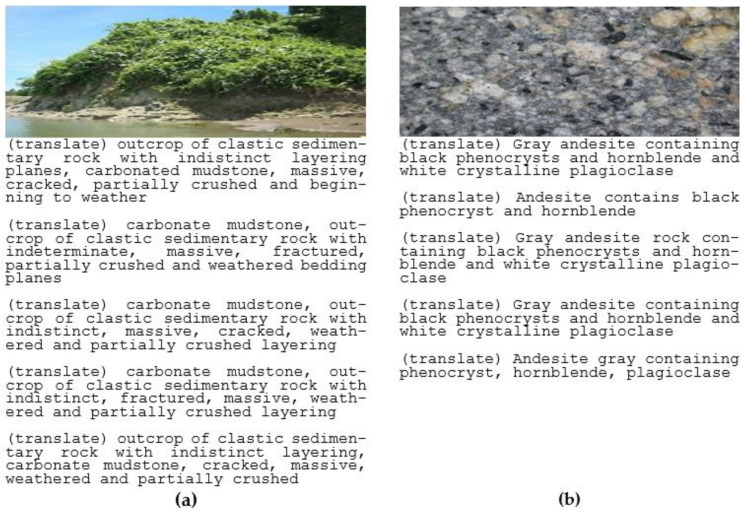
Geological rock images and their captions translated into English: (**a**) captions for the geological image of sedimentary rocks and translated into English; (**b**) captions for geological image on andesite contents and translated into English.

After completing the processing of both parts, the process continues to add both units to be one pairwise vector feature. In Figure 4, there is an operation ADD (X1, X2). This means that both units were flattened from the image and text extraction to create one flattened vector feature. After that, the last process was to operate the SoftMax function to acquire the word classification. The word classification process aligns the predicted word with the image feature.

### 2.4. Word Embedding

To provide vector value embedding, we used Word2Vec as a word embedding model to provide a map of values of the words. Word2vec was introduced by Thomas Mikolov and consists of two processes: continuous bag of words (CBOW) and continuous n-skip gram. Each process has a unique task in handling a word. CBOW acts as a neural network process that gives a probability value and selects the higher probability as a candidate value. On the other hand, the continuous n-skip gram process takes the current word as the input and tries to accurately predict the words before and after this current word [54]. This study used 100 dimensions for each word and there was a 306 × 100 vector space for all words.

## 3. Results

This section reports on the experiments’ achievement and is separated into two subsections: dataset and experiments. We used Google Collaboratory Pro, Python version 3.6, TensorFlow, a GPU, and 25 GB of RAM for the experiments. The GPU used by default Google Collaboratory is NVIDIA T4 or P100.

### 3.1. Dataset

We started by collecting the images and carrying out the data preprocessing. Following the proposed pipeline model in Figure 3, the first step was to reduce the image into 224 × 224 pixels with RGB color. As the input in the CNN, all images were set to 32 channels in the first convolution process. 

We collected 1297 images of geological rocks and divided them into two datasets. The training dataset was 1001 images and the validation dataset was 296 images. In addition, we added five captions for each image and acquired a caption from a geologist for each image as a geological corpus. The caption was completed by the geologist following the guidelines of writing lithology descriptions and arranged into the names of the rock, color, and dominant rocks [55]. 

Figure 4 shows examples of images from the dataset and their captions. The captions were written in the Indonesian language and we translated the text to make the captions clear. This study was focused on developing a model in the Indonesian language, because of its usefulness for further study. The translation is included for the purpose of making the paper easy to read. 

### 3.2. Experiments

The proposed models were assembled from reengineering the VGG16 and our model called CNN-n. The choice of the name CNN-n is based on the experiments, which consisted of several layers or shallow learning. Our model was divided into CNN-A, CNN-B, CNN-C, and CNN-D sub-models. The CNN-n model was introduced from the image classification results regarding rock types [56]. CNN-D using conv (32, 5) and conv (64, 5) produced a larger number of output parameters than the CNN-A, CNN-B, and CNN-C models. Simonyan and Zisserman stated that the filter plays an important role in extracting an image [57]. Filters are square matrices with odd numbers, such as 1 × 1, 3 × 3, 5 × 5, and 7 × 7 [57]. In several studies on image extraction, scholars used many 3 × 3 filters. This particular filter proved effective in extracting the image and providing a feature map for recognizing the image [53,58].

**Figure 5:**
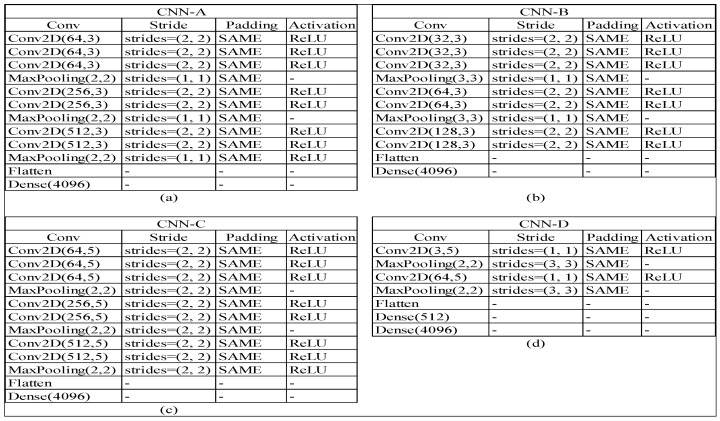
Architectures of proposed CNN models. (**a**) CNN with seven layers convolutional and uses three layers of MaxPooling, (**b**) CNN with seven layers convolutional and two layers of MaxPooling, (**c**) same as (**a**) but different size of MaxPooling, (**d**) CNN with two layers convolutional, two MaxPooling layers, and two fully connected (FC) layers.

Figure 5 shows several architectures from our re-engineered CNN models. We obtained outputs for VGG16, CNN-A, CNN-B, CNN-C, and CNN-D of 134,260,544, 12,746,112, 2,397,504, 20,482,432, and 104,867,300, respectively. These values were created from the flattening unit multiplied by the defined output unit. For instance, CNN-D had 104,867,300 parameters; for the CNN-D architectures in Figure 5d, the last fully connected (FC) layer was 50 flattened units, 512 dense units, and 4096 FC units. The parameter size is created by multiplication among the flattened units, dense units, and FC units. Thus, the parameter output was 104,867,300. The 50 flattened units are array units that provide output from the convolutional process after the MaxPooling layer and ReLu functions. 

We delivered the duplicate operation pruning for each model at the last layer of unit classification. These actions were taken because we needed the weight of the units for the subsequent process. The process continued to concatenate operations between FC units from the CNN and LSTM units to gain a predicted word. This model follows the likelihood function $L\left(\theta \right)=\overset{n}{\underset{i}{\prod }}p\left(w_{1},w_{2},\ldots w_{n},\;I|\theta \right)$, where w stands for previous words, I is an image region, and θ is a predicted word. 

**Figure 6:**
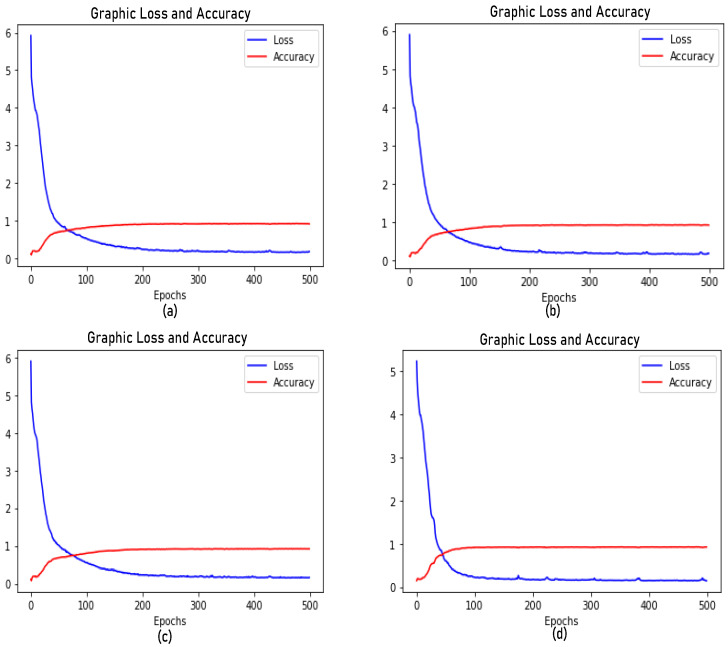
Accuracy and loss graphs of the proposed models: (**a**) CNN-A, (**b**) CNN-B, (**c**) CNN-C, and (**d**) CNN-D. The experiments used 500 epochs as a training measure. The graphs show that each model needed just below 100 epochs to reach its best accuracy.

Figure 6 shows the accuracy curve for each CNN model and depicts the comparison between loss and accuracy. We observed that the accuracy increased at 80 epochs. Our CNN-A, CNN-B, CNN-C, CNN-D, and VGG16 architecture had accuracies of 0.9137, 0.9148, 0.9178, 0.9206, and 0.9228, respectively. Figure 6 presents how much the experiments were influenced by the number of CNN layers and parameters in the domain under study. The curves depend on the receptive field and channel settings.

Table 1 shows a comparison of the training parameters for each of the CNN architectures. The VGG16 model had 134,260,544 training parameters for image extraction and 4096 dense units. However, CNN-B and CNN-D had the same processing time. In order to achieve a sentence that could be adequately read, we generated sentences using a beam search algorithm [59]. 

Table 2 presents several proposed models constructed with the CNN, LSTM, and bi-LSTM. For the baseline model, we prefer VGG16 + bi-LSTM + one hot vector [61] and several models, such as ResNet [14] and InceptionV3 [62]. Thus, we re-engineered several models and embedded using Word2Vec.

We employed the BLEU score to measure the precision of candidate captions and reference captions. The measured BLEU scores for 500 epochs are shown in Table 2 using the validation dataset. Supposing the translation results are identical to the reference sentence and have the same length of words, then the BLEU score is 1.00. This study used a pre-trained model, Word2Vec, to obtain a feature vector for every single vocabulary word from the text processing. Using the proposed models, we achieved 306 × 100 feature dimensions and then used a flattening operation to gain 256 units. We used the Indonesian corpus ourselves to make a feature vector that related to geological captions. Table 2 shows that the B@1 score was 0.6463, 0.7012, 0.7620, and 0.5620 for CNN-A, CNN-B, CNN-C, and CNN-D, respectively. These scores were acquired from the following BLEU formula: $\mathrm{BLEU}={BP*\;e}^{\left(\sum_{i=1}^{N}W_{n}\log P_{n}\right)}$ with $BP=\{\begin{array}{c} 1\\ e^{\left(1-r/c\right)} \end{array}\;\begin{array}{c} ,\;\mathrm{if}\;c>r\\ ,\;\mathrm{if}\;c\;\leq r \end{array}$. 

This study leveraged the precise parameter metric, which was introduced by [60]. The BLEU score emphasizes precise captions using the N-gram. Proper and grammatical order is a significant requirement for clear meaning. The BLEU score measures how much a caption is similar to a geologist’s descriptions.

**Figure 7:**
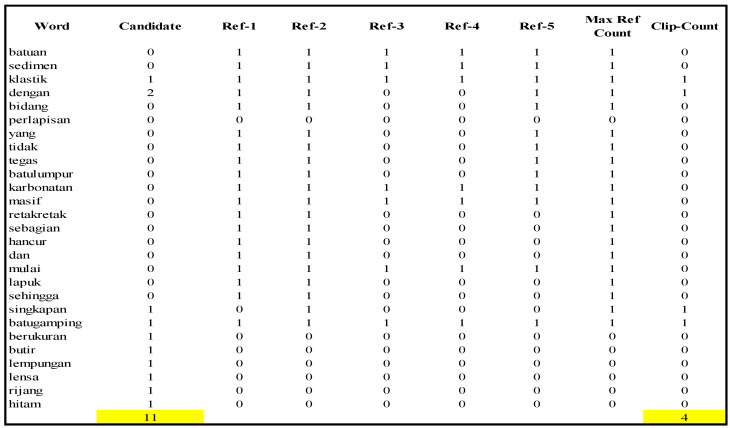
Collecting tokens taken from reference and candidate captions in Indonesian. Candidate is the number of words from the caption generation, ref-n is the number of words that have a similar word as the candidate, Max ref count is a value from selecting Max (candidate, ref-n), and clip_count is a value of Min (candidate, Max ref count). The yellow color is sum of candidate and sum of cli-count.

Here, we have written an illustration for measuring a caption. For instance, we acquired a caption, such as “singkapan batugamping klastik dengan berukuran butir lempungan dengan lensa rijang” in Indonesian. We calculated the BLEU score by the following algorithm [60]:

1. Tokenize each caption $\left(w_{1},w_{2},\ldots ,w_{n}\right)$;
2. Calculate the variable “count” and “clip_count” from the reference token and candidate token, see Figure 7;
3. Compute precision modification with formula $p_{n}$ $=\frac{clip\_count}{word\_length}=\frac{4}{20}=0.2$,
4. If length of candidate <= reference, calculate brevity penalty (BP) with $BP=e^{\left(1-\frac{r}{c}\right)}=e^{\left(1-\frac{20}{2}\right)}=0.442$, else *BP* = 1;
5. Calculate BLEU-1 and the result can be seen in Table 2.

Figure 7 express the collection of vocabulary for each reference. These tabular shows the simulation counting of every single word in the caption. Candidate column show how many words that have similar with the reference. Beside that, ref-1 trough ref-4 is the calculating number of words to compare with the candidate.

**Figure 8:**
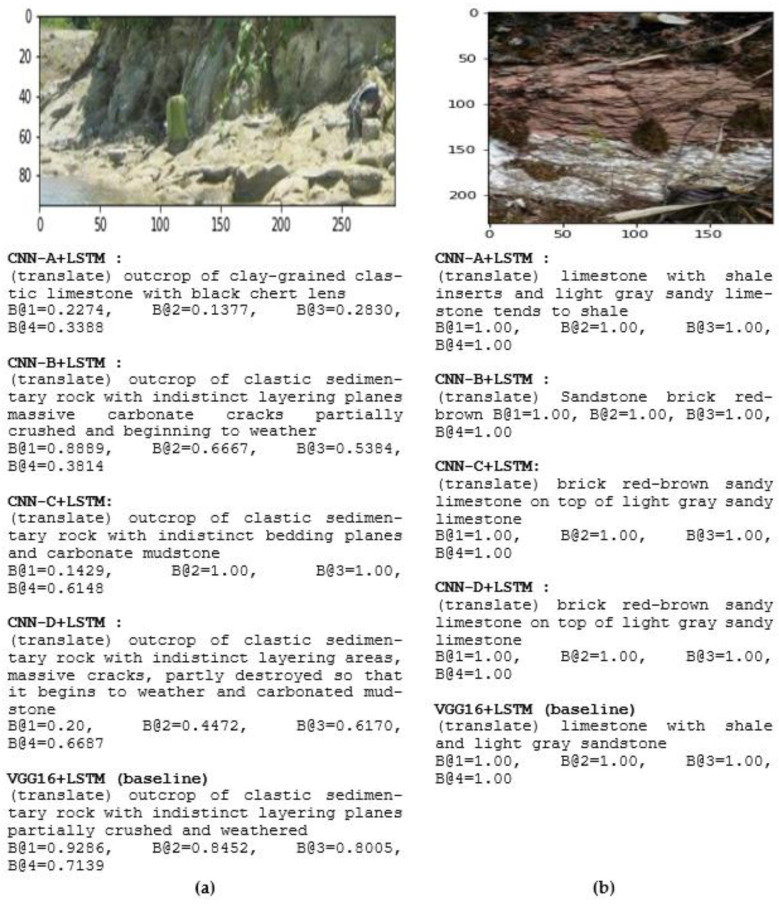
Examples of generated captions for geological captioning, translation into English. B@N is the BLEU score. (**a**) Sedimentary rock image; (**b**) limestone image on a different surface.

Figure 8 shows an instance of the captioning from our training models. We validated the training model using dataset validation, as shown in Figure 8. Different layers, filters, and training parameters caused different results. Varying the parameters supports the creation of an accurately predicted word that aligns with the image area [2]. It is a point of discussion because, in CNN operation, every mapping process results in a new block value that is smaller than before. Reducing the H × W × D of the CNN block avoided overfitting [5].

We compared the generated captions with ground-truth captions. Figure 8a compares five geologist references as follows: Ref-1: singkapan batuan sedimen klastik dengan bidang perlapisan yang tidak tegas, batulumpur karbonatan masif retakretak sebagian hancur dan mulai lapuk—(translation) outcrop of clastic sedimentary rock with indistinct layering, massive fractured carbonate mudstone partially crushed and beginning to weather; Ref-2: singkapan batuan sedimen klastik dengan bidang perlapisan yang tidak tegas masif retakretak sebagian hancur sehingga mulai lapuk dan batulumpur karbonatan—(translation) outcrop of clastic sedimentary rock with indistinct layering areas, massive cracks, partially destroyed so that it begins to weather and carbonate mudstone; Ref-3: singkapan batuan sedimen klastik dan batulumpur karbonatan—(translation) outcrop of clastic sedimentary rock and carbonate mudstone; Ref-4: batulumpur karbonatan dan singkapan batuan sedimen klastik—(translation) carbonate mudstone and outcrop of clastic sedimentary rock; Ref-5: singkapan batuan sedimen klastik dengan bidang perlapisan yang tidak tegas dan batulumpur karbonatan—(translation) outcrop of clastic sedimentary rock with indistinct bedding planes and carbonate mudstone.

In Figure 8b, we compared five geologist captions by referring to the ground truth: Ref-1: batugamping dengan sisipan serpih—(translation) limestone with shale insert; Ref-2: batugamping pasiran berwarna merah bata/kecoklatan—(translation) brick red/brown sandy limestone; Ref-3: batugamping pasiran berwarna merah bata/kecoklatan menindih batugamping pasiran berwarna kelabu cerah—(translation) brick red/brown sandy limestone on top of light gray sandy limestone; Ref-4: Batugamping bersisipan serpih dan batugamping pasiran berwarna kelabu cerah—(translation) limestone with shale inserts and light gray sandy limestone. Ref-5: Batugamping bersisipan serpih dan batugamping pasiran berwarna kelabu cerah cenderung menyerpih—(translation) limestone with shale inserts and light gray sandy limestone tends to shale.

## 4. Discussion

There are several discussions to be presented. The objective was to find the best captions by obtaining a high BLEU score. We followed the proposed method as a strategy for observing the object in the background [60]. The experiments proved to have different outcomes. A change in the layers can cause various outcomes, such as in the resulting caption, training parameters, and time. An event in the convolution process and size filtering can result in different feature maps. The number of channels the CNN has influences the feature maps’ colors, shapes, and sizes. We set a significant number of channels, which provided opportunities for various gradations of colors. On rock images, observing the color is necessary to distinguish the content of the rocks; even if the rocks have a similar color, the content is probably different [53]. 

VGG16 successfully identified the object and predicted the classification [63,64,65]. Meanwhile, our models successfully classified the rock object within an efficient time, particularly CNN-B and CNN-D [66]. Table 1 shows that VGG16 is suitable for our rock images, but the model has a longer processing time compared to the CNN-B and CNN-D models. 

Table 3 compares the accuracy and loss of the models. The results showed that VGG16 achieved the best accuracy and that several layers and structure processes applied to the CNN were robust in differentiating color. 

The theoretical concept of VGG16 tries to extract the image from deeper layers and provide outcomes with various features [65]. In image recognition, the detection of similar features is an essential task to achieve the best accuracy. In our problem domain, if the image features concatenate with text features, then many pairwise mistakes can occur and cause the wrong predicted word. Many feature map differences cannot guarantee that the captions will be optimized. The spread of various colors causes a bias when the object area pairs with the sentence. The effect that many features create causes probable bias in predicting words. We used a limited object in our domain, which is why the results of pairwise testing a sequence of image descriptors and feature vector text is important. 

LSTM, as a word generator, was chosen as the language model because of its ability to separate outcomes into three gates. The LSTM generates words based on previous words by relating to the corpus and selecting the best probability word from many produced words. The previous word makes the LSTM machine more powerful in predicting the next word [67]. This study found error captions for the VGG16+word2vec+LSTM model when predicting a word. Simonyan models have successfully generated a word when the object is a person, an animal, a car, etc. [57]. In contrast, the models experience an error in predicting a word when the object appears in the background.

The likelihood function $\log P\left(w_{t}|w_{1:t-1},I;\theta \right)$ is important for making a true predicted word when generating a word. Re-engineering of the log-likelihood function will result in different captions. We acquired a loss value of 0 ≤ $\sum_{I,S\epsilon X}\sum_{t=1}^{\left|s\right|}\log P\left(w_{t}|w_{1:t-1},I;\theta \right)$≤ 0.1358. This means that the function was successful in maximizing the detection of images and predicting the words by minimizing the residual loss values [68]. 

**Figure 9:**
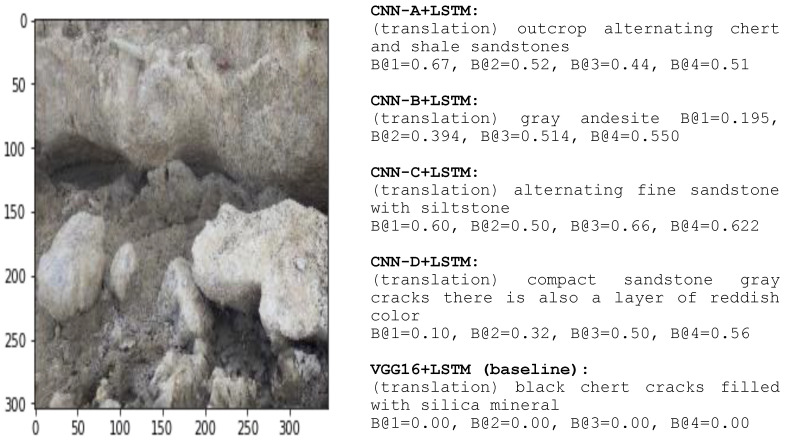
An error created by the baseline model, producing a different rock type. The dominant rock in the image is sandstone, whereas the baseline produced chert and CNN-B produces gray andesite.

Figure 9 shows an error caption and compares VGG16 + Word2Vec + LSTM to our models [69]. This study successfully presented an error result from the baseline. It proves that many CNN layers do not always acquire the best outcome; indeed, the result is sometimes a mistake. The image in Figure 9 is of sandstone. Nevertheless, the baseline presented a mistake caption of chert. Many parameters produced the feature that caused VGG16 difficulty to pairwise between the word and the feature maps. Geological rock images always show a variety of color within the rocks and color differentiation is necessary to separately identify the rocks. 

Some images could not be adequately classified by VGG16, thus, resulting in a low BLEU score. However, it is understandable to assume that VGG16 is accurate because it has a deeper layer than our models. The impact of this is that many feature maps eventually become biased when rephrasing image feature extraction and word embedding. It is known that image feature extraction becomes decisive regarding feature identification. The number of CNN layers can convey the success of feature extraction and many receptive field channels supply more space for feature assortments [70,71]. It can help to recognize objects to a certain degree in an image. Meanwhile, the pooling layer also helps the feature map values to avoid overfitting.

This study shows a need to recognize and analyze the image relating to captions by using image preprocessing, such as reducing image size. In the caption, it is necessary to pay attention to the text marks when reading. Sometimes, captions use the “-” sign to make adjectives or derivatives of rock types. In text preprocessing, the text is cleaned by removing the stop word, sign, number, and symbols. Figure 3 shows the word vectorizing using Word2Vec as a feature text. The difference between one hot vector and word2vec lies in the matrix values. One hot vector consists of a 0 or 1 value and has a length the same as the defined word length [28,31]. On the other hand, word2vec creates a decimal value with the defined length and dimensions [25,69].

Regarding the process, the annotation of the rocks was accompanied by their properties, such as carbonate mudstone and clay sandstone [72]. The classification and interpretation of images created a caption based on feature maps and the text feature. This scheme is an essential part of captioning because this research’s target was a caption similar to the geologist’s description [53].

## 5. Conclusions

Our models outperform the architecture of the baseline model. A CNN (32,5) with a 5 × 5 filter and 32 channels produced a meaningful caption. The metric of the model was directed more toward the precision of the caption than accuracy. The accuracy is just needed to measure the image classification and how similar the factual feature map extraction compares with the actual feature maps. The experiments proved that shallow layers effectively solved our domain problem. Our proposed CNN-A, CNN-B, CNN-C, and CNN-D models used BLEU score methods for the N-gram. The BLEU scores achieved for B@1 were 0.5515, 0.6463, 0.7012, 0.7620, and 0.5620, respectively. B@2 showed scores of 0.6048, 0.6507, 0.7083, 0.8756, and 0.6578, respectively. B@3 had scores of 0.6414, 0.6892, 0.7312, 0.8861, and 0.7307, respectively. Finally, B@4 showed scores of 0.6526, 0.6504, 0.7345, 0.8250, and 0.7537, respectively. The CNN-D architecture encouraged our model to produce a high B@4 score of 0.8250 but it had a time deficiency. The BLEU score measurement was dependent on precision and word embedding. The combination of the CNN and Word2vec embedding increased the speed and produced precision words. Construction of the caption using the beam search supported the creation of proper sentences for the caption. There are several considerations for building the architectures, such as the optimum number of layers, the precise ReLu function, a suitable SoftMax function, and an ADAM optimizer tuned to acquire good results. On the other hand, the accuracy score is used to measure how precisely the image detection matches the image references. The metrics used for measuring the success of image detection are similar for captioning.

Relating to the results, we discovered several challenges for future research. This study did not just find layers, filters, strides, and pooling methods but also proposed language generators. Language models, such as structured language and paraphrasing models, are important subjects of research. Assembling captioning based on geological sentence arrangement, geological sentence representations, and assembling words by geological sentence tagging is a challenging topic. In all captioning models, the target is word precision, which is an indicator of success when generating a caption, with a high BLEU score or other language metric.

## Figures and Tables

**Table 1:** Training parameter comparison. ADAM optimizer with lr = 0.0001, MaxPooling, ReLu activation, and 4096 flattened units employed for tuning parameters.

Model	Layer	Filter	Parameters	Size	Time(s)
VGG16	16	1 × 1, 3 × 3	134,260,544	224 × 224	1845
Ours_CNN-A	7	3 × 3	12,746,112	224 × 224	1860
Ours_CNN-B	7	3 × 3	2,397,504	224 × 224	1230
Ours_CNN-C	7	5 × 5	20,482,432	224 × 224	1885
Ours_CNN-D	2	5 × 5	104,867,300	224 × 224	1230

**Table 2:** Comparison of BLEU-N scores. Results gained from experiments using the testing dataset and executed using the baseline and proposed models.

Model Caption	BLEU-1 (Unigram)	BLEU-2 (bi-gram)	BLEU-3 (3-gram)	BLEU-4 (4-gram)
VGG16 + LSTM + word2vec	0.5516	0.6048	0.6414	0.6526
ResNet50 +LSTM + Word2Vec	0.3990	0.3830	0.4030	0.3440
InceptionV3 +LSTM + Word2Vec	0.3320	0.3120	0.3300	0.2730
Ours_CNN-A + LSTM + word2vec	0.6464	0.6508	0.6892	0.6504
Ours_CNN-B + LSTM + word2vec	0.7012	0.7083	0.7312	0.7345
Ours_CNN-C + LSTM + word2vec	0.7620	0.8757	0.8861	0.8250
Ours_CNN-D+ LSTM + word2vec	0.5620	0.6578	0.7307	0.7537
CNN + LSTM + One-Hot, adapted from [2], Copyright 2015, Karpathy et al.	0.3990	0.3830	0.4050	0.3470
InceptionV3 + LSTM + One-Hot [14]	0.3760	0.3410	0.3540	0.2960
ResNet50 + LSTM + One-Hot [14]	0.4030	0.3920	0.4150	0.3590

**Table 3:** Comparison of the accuracy and loss between our models and the baseline.

Evaluation	CNN-A	CNN-B	CNN-C	CNN-D	VGG16
Accuracy	0.9137	0.9148	0.9178	0.9206	0.9228
Loss	0.1763	0.1764	0.1638	0.1457	0.1464
